# Comprehensive diagnostic and therapeutic approaches to uterine teratoma in postmenopausal women: a case study and review

**DOI:** 10.3389/fonc.2024.1458187

**Published:** 2024-12-11

**Authors:** Wenwei Pan, Jun Lan, Zihao Tang, Taikang Li, Xinping Ma

**Affiliations:** ^1^ Department of Obstetrics and Gynecology, Dongguan Maternal and Child Health Care Hospital, Dongguan, China; ^2^ Dongguan Maternal and Child Health Care Hospital, Dongguan Key Laboratory of Female Reproductive Health, Dongguan, China

**Keywords:** postmenopausal women, uterine teratoma, ultrasonography, oncological imaging, hysteroscopy surgery, pathological diagnosis

## Abstract

**Background and objective:**

Uterine teratomas are extremely rare in postmenopausal women and pose significant challenges in diagnosis and treatment. This study reports a case of a 54-year-old postmenopausal woman diagnosed with a uterine teratoma during a routine gynecological ultrasound. The study aims to explore the diagnosis and treatment of uterine teratomas in postmenopausal women through detailed imaging assessment and hysteroscopy surgery, aiming to enhance the understanding of this rare uterine tumor and improve patient treatment outcomes and quality of life.

**Methods:**

This study utilized hysteroscopic surgery to excise a mass initially suspected to be a uterine fibroid and performed pathological analysis. The analysis revealed the presence of fat and hair, confirming the diagnosis of a mature benign uterine teratoma. Additionally, the study includes a literature review summarizing the clinical characteristics, diagnostic methods, and treatment strategies for uterine teratomas.

**Results:**

Hysteroscopic surgery effectively identified and removed the complex uterine teratoma. Pathological analysis confirmed the teratoma contained various mature tissues, including neural tissue, bone, and cartilage. Comparative analysis of imaging features with surgical outcomes helped propose more precise diagnostic criteria and treatment guidelines.

**Conclusion:**

Hysteroscopy surgery plays a crucial role in diagnosing uterine teratomas in postmenopausal women and showcases its advantages of minimal trauma and quick recovery in treatment. In addition, the literature review emphasizes the diagnostic challenges of uterine teratomas in clinical practice, particularly in expanding clinical awareness of the differences between premenopausal and postmenopausal presentations. It also summarizes the diagnostic and treatment strategies for rare uterine masses, contributing to the improved recognition of these rare cases.

## Highlights

- The study focuses on exploring the clinical presentation and diagnostic challenges of uterine teratoma in postmenopausal women.
- The main results show that hysteroscopy surgery can effectively identify and remove complex uterine teratoma.
- Various mature tissues within the mass provide important diagnostic clues for misdiagnosis.
- This study summarizes rare uterine masses’ diagnostic and treatment strategies.

## Introduction

Teratomas are the most common germ cell tumors (GCT) in young people, especially in the testicles and ovaries (1). These tumors typically contain tissues from different embryonic layers, such as skin, hair, muscle, and bone (2). Although the discovery of uterine teratomas in postmenopausal women is rare, their diverse clinical manifestations and treatment strategies make them a challenge in gynecologic tumor diagnosis and treatment (3, 4). The hormonal changes in postmenopausal women may affect the biological behavior of the tumors, increasing the complexity of diagnosis and treatment (5–7).

Cystic teratomas are usually easily preliminarily diagnosed through ultrasound examination, and confirmation is obtained through histopathology after surgery (8). Although these tumors are mostly benign, the risk of malignant transformation should not be ignored, especially in older women, with common transformations to squamous cell carcinoma and adenocarcinoma (9). Ectopic teratomas are rare in midline areas of the body, such as between the sacrum and coccyx, head and neck regions, posterior peritoneal spaces, mediastinum, and intracranial spaces (10, 11).

Hysteroscopy surgery, with minimal trauma and quick recovery advantages, is important in treating gynecologic tumors (12–14). Compared to traditional open surgery, hysteroscopy can remove tumors through smaller incisions, reducing intraoperative bleeding and postoperative pain and shortening hospital stay (15–17). In one case of a 54-year-old female with uterine mature teratoma in this study, the initial misdiagnosis suggested a submucosal fibroid or endometrial polyp with epithelial metaplasia. Further tumor imaging revealed specific enhancement features of the mass (18).

Teratomas originating from the uterus are extremely rare in medical literature, with very limited reports since they were first reported in 1929. In hysteroscopy surgery, a lesion initially thought to be a uterine fibroid turned out to be a mature benign uterine teratoma containing fat and hair, a highly unusual finding (19–21). The final pathological analysis confirmed that the teratoma contained fat and hair and various mature tissues such as neural tissue, bone, and cartilage.

This study aims to explore the diagnosis and treatment of postmenopausal women uterine teratoma through detailed imaging evaluation and hysteroscopy surgery. We hope to clarify the relationship between imaging features and surgical outcomes through comparative analysis and propose more precise diagnostic criteria and treatment guidelines. Additionally, this study will summarize existing clinical treatment experiences through a literature review to provide a basis for future clinical practice. Ultimately, we hope this study will enhance doctors’ understanding of the diagnosis and treatment of uterine teratomas in postmenopausal women, improve patient treatment outcomes and quality of life, and have significant educational and clinical significance.

## Case presentation

**Figure 1:**
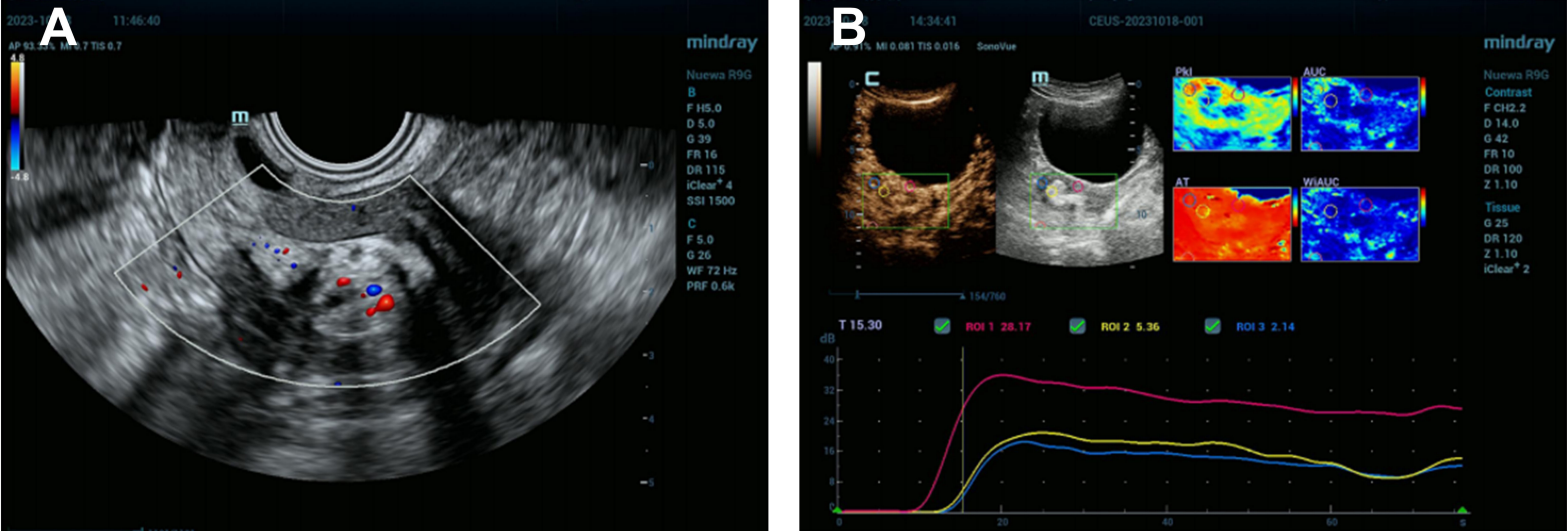
Uterine Mixed Echogenic Lesion Revealed by Comprehensive Ultrasonography and Enhanced Imaging Studies. **(A)** Preoperative transvaginal ultrasound images showed a normal uterine morphology with a uniformly thin endometrium. A mixed-echo lesion was observed within the uterine cavity, measuring 30 mm × 17 mm × 21 mm. The images suggested a submucosal leiomyoma with metaplasia or an endometrial polyp. No obvious masses were detected in the bilateral adnexa. The left ovary measured 12 mm × 7 mm, and the right ovary measured 12 mm × 6 mm. **(B)** Enhanced tumor image displaying late high enhancement relative to the muscle layer, followed by late low enhancement, indicative of submucous leiomyoma with metaplasia.

This case report details the clinical diagnostic process of a 54-year-old postmenopausal woman. The patient’s medical history indicates G3P2A1, with a history of one vaginal delivery, one cesarean section, and one spontaneous abortion. Despite the absence of significant symptoms, a uterine mass was incidentally discovered during a routine transvaginal ultrasound examination, leading to further evaluation at the hospital. The utilization of ultrasonography as an initial diagnostic tool, owing to its non-invasive nature, efficiency, and cost-effectiveness, provides real-time imaging assisting in the observation of uterine internal structures and preliminary assessment of the nature of the mass, including its size, location, and echogenic characteristics. In this case, ultrasonography revealed a mixed echogenic lesion measuring 30mm × 17mm × 21mm, displaying heterogeneous echogenicity internally, possibly comprising different tissue components of solid and liquid nature. Such a mixed echogenicity is commonly seen in benign conditions, such as submucous leiomyoma with metaplasia or endometrial polyp, leading to a preliminary diagnosis leaning towards these two possibilities (**Figure 1A**). In addition, the ultrasound examination revealed the condition of the ovaries and adnexa: the left ovary measured 12 mm × 7 mm, the right ovary measured 12 mm × 6 mm, and no obvious masses or other abnormalities were observed in the bilateral adnexa (**Figure 1A**).

**Figure 2:**
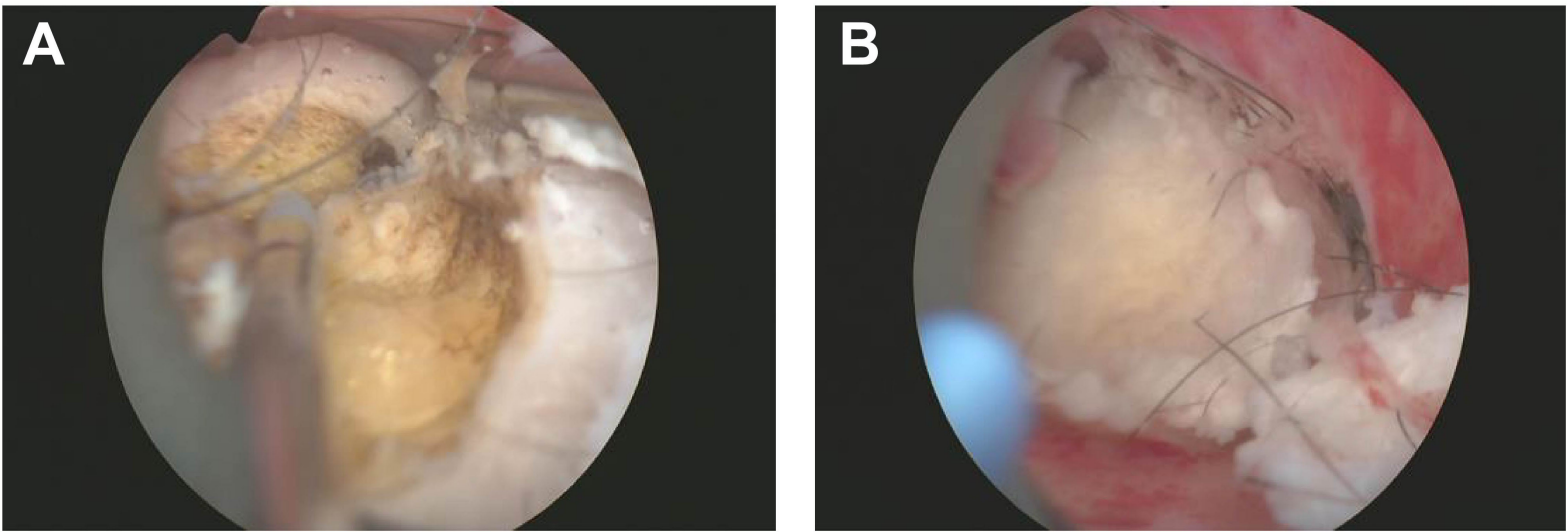
Uterine Teratoma Containing Fat and Hair Detected by Hysteroscopy. This image illustrates the successful resection of a teratoma from the uterine wall during hysteroscopy surgery. **(A)** Initial exposure to the teratoma revealed yellow adipose tissue, indicating the presence of fat components within the lesion. **(B)** Further dissection of the mass exposed white fibrous tissue and other complex elements, demonstrating the teratoma's heterogeneous composition and irregular surface.

Considering the location and nature of the lesion, more in-depth tumor imaging studies were conducted. Results from these examinations indicated that the mass exhibited high enhancement in the early phase but transitioned to low enhancement in the later phase, a presentation possibly congruent with submucous leiomyoma with metaplasia (**Figure 1B**). Based on these findings, the patient was scheduled for hysteroscopy surgery for the excision and further analysis of this lesion. During the hysteroscopy examination, the surgeon first dilated the cervix to insert the hysteroscope, a thin and bright tube equipped with a camera, into the uterus. Once inside the uterus, saline solution was injected to expand the uterine cavity for better visibility. Real-time camera transmission enabled the surgeon to locate and assess the leiomyoma-like mass. Using specialized instruments passed through the hysteroscope, the surgeon meticulously dissected the mass from the uterine wall. Initially suspected to be a typical fibroid, the texture and content of the mass—revealing fat and hair—indicated that it was, in fact, a teratoma (**Figures 2A, B**). Continuing with minimally invasive surgical techniques, the surgeon carefully separated the teratoma from surrounding tissues. Due to the presence of various tissue types within the teratoma, including fat, hair, and possibly bone or tooth-like structures, precise surgical maneuvers were required to avoid damaging the uterine wall or causing excessive bleeding.

**Figure 3:**
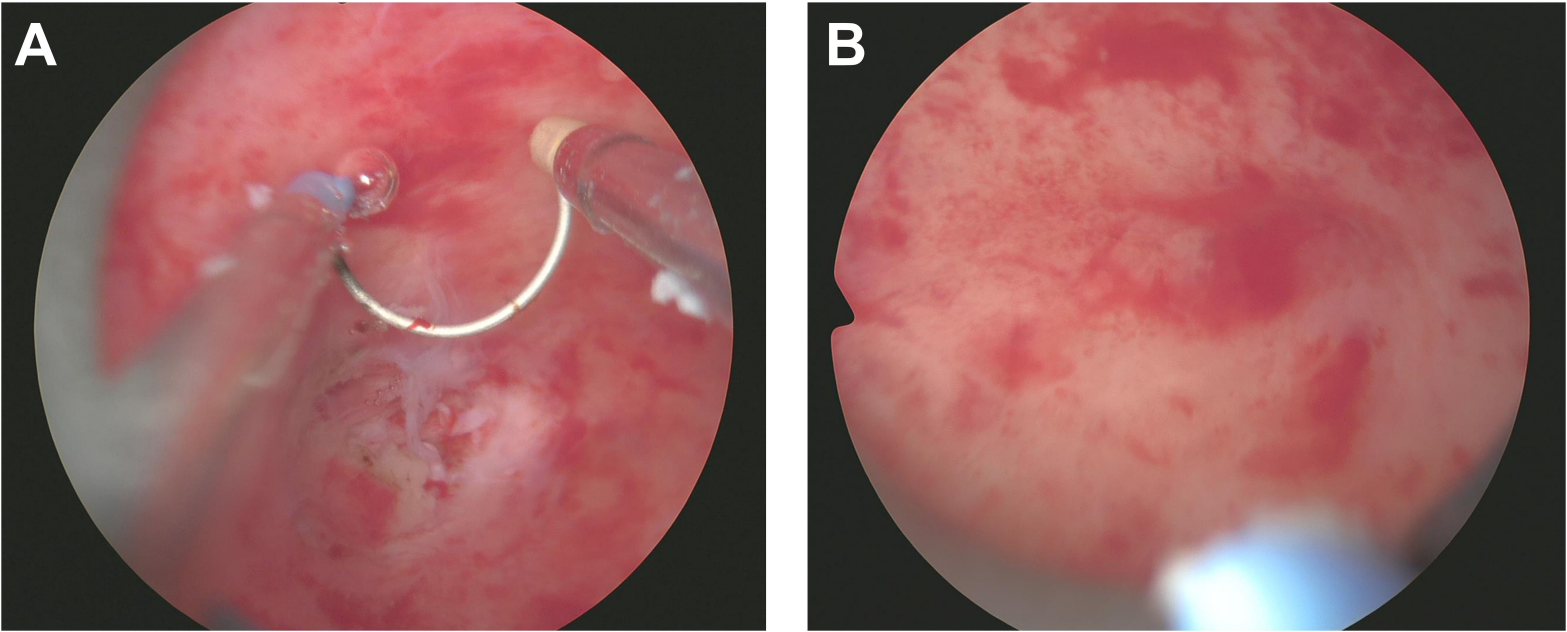
Resection of Uterine Teratoma during Hysteroscopy Surgery. **(A)** This image demonstrates the meticulous surgical procedure under hysteroscopy, where the surgeon delicately separates the teratoma from the uterine wall, containing fat, hair, and possibly bone or tooth-like structures. **(B)** Post-complete excision of the mass within the uterus, the uterine wall appears smooth and without evident abnormalities, leaving only minimal incision marks in the surgical area.

**Figure 4:**
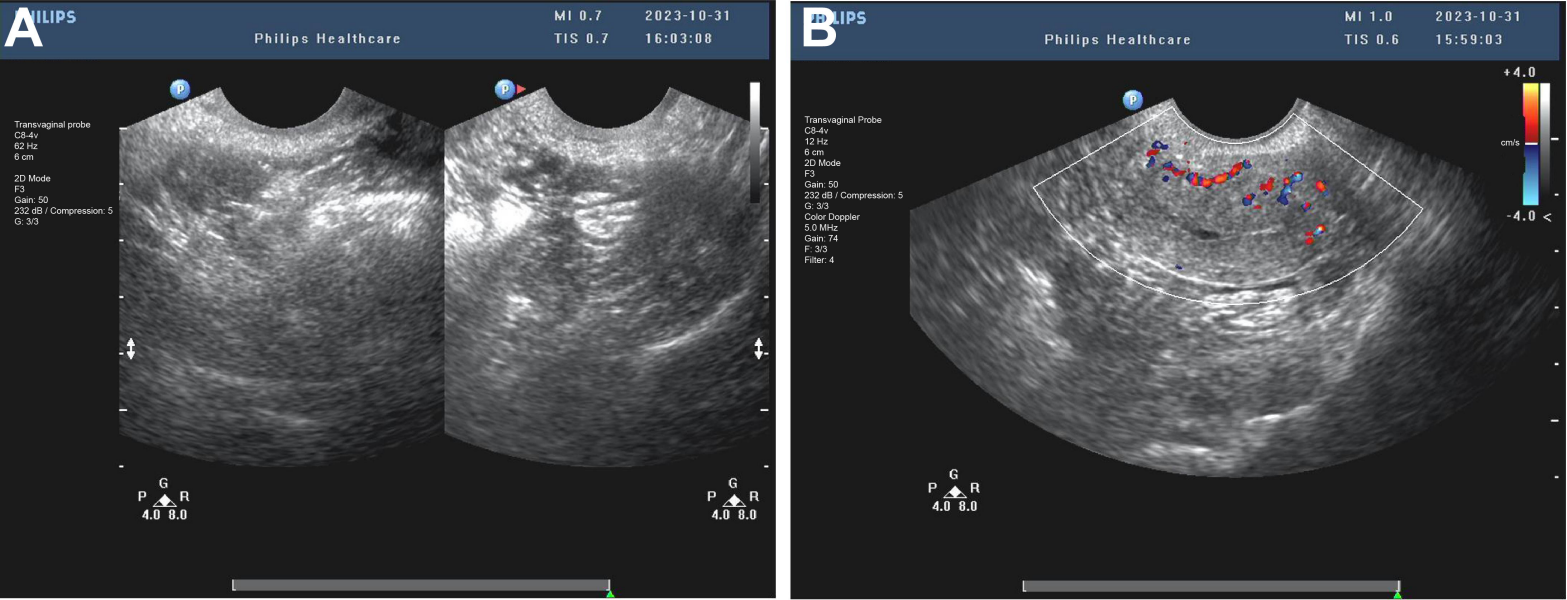
Postoperative transvaginal ultrasound assessment of the uterus and adnexa. **(A)** The postoperative ultrasound image shows a retroverted uterus of normal size, with a length of 36 mm, an anteroposterior diameter of 25 mm, and a transverse diameter of 37 mm. **(B)** The postoperative ultrasound shows no abnormal masses in the bilateral adnexa. The left ovary measures 18 mm × 11 mm, and the right ovary measures 20 mm × 11 mm, indicating that the surgery did not cause any ovarian or adnexal lesions.

Throughout the excision process, the surgeon constantly adjusted the instruments’ angles and positions to ensure complete mass removal of the mass in one piece while preserving uterine integrity (**Figure 3A**). After complete excision of the mass, the hysteroscopic view became clear. The excision area was thoroughly examined to confirm the absence of residual foreign bodies or abnormal tissues. Saline solution was re-injected to cleanse the surgical site and evaluate for any bleeding points requiring attention. Following these steps, the hysteroscope was slowly withdrawn. Post-resection images revealed a smooth uterine wall without any obvious abnormalities. The mass was entirely excised, and the surgical area appeared normal, with only minor incision marks that typically heal naturally within a few weeks (**Figure 3B**). Postoperative management focuses on monitoring for bleeding or infection, with the excised tissue sent to pathology for final analysis. After the surgery, the patient underwent postoperative ultrasound to evaluate recovery. The examination showed a retroverted uterus of normal size, with a uterine length of 36 mm, anteroposterior diameter of 25 mm, and transverse diameter of 37 mm. No abnormal masses were found in the bilateral adnexa (**Figure 4A**). The ovarian volumes were slightly larger than preoperatively: the left ovary measured 18 mm × 11 mm, and the right ovary measured 20 mm × 11 mm (**Figure 4B**). These results indicate that the surgery did not cause adnexal lesions or ovarian abnormalities, and the patient was recovering well. Postoperative management focuses on monitoring for bleeding or infection and ensuring no other complications. The extracted tissue was sent for pathological analysis for final evaluation.

**Figure 5:**
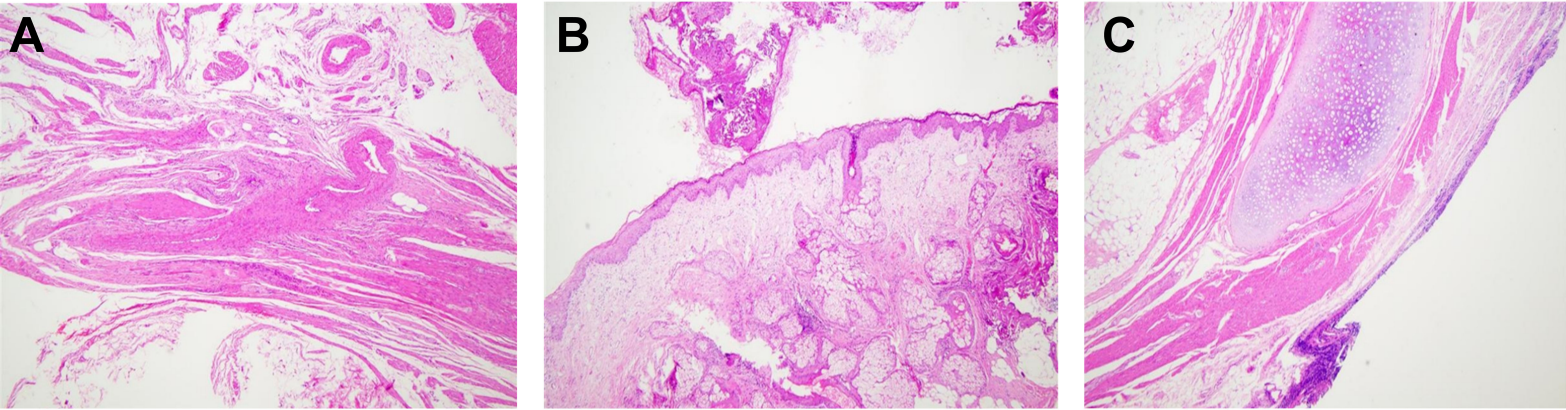
Histological Characteristics of Mature Uterine Teratoma. **(A)** Cross-section stained with hematoxylin-eosin showing the histological features of a mature cystic teratoma. **(B)** Sebaceous gland structures within the tumor. **(C)** Detailed view of skin appendages within the tumor (magnification ×10).

Further pathological analysis revealed that the lesion was of mixed cystic and solid nature, containing various tissue components such as hair, bone, fat, and cartilage. Microscopic examination identified structures of skin appendages, fat tissue, neural tissue, and cartilage within the lesion, confirming that it was a mature benign uterine teratoma. Teratomas are tumors containing various mature tissue types, an exceedingly rare occurrence in the uterus. In this case, the teratoma contained fat tissue, hair, neural tissue, bone, and cartilage, among other tissues (**Figure 5A**). The presence of these tissues further validated the teratoma diagnosis, as these tumors typically consist of tissues from all three embryonic layers.

Additionally, histological examination revealed squamous epithelium-carrying hair, typically found in the skin, underscoring the tumor’s heterogeneity and complexity within the uterine teratoma (**Figures 5B, C**). Further tissue analysis depicted well-differentiated neural tissue within the teratoma, showing no signs of malignant transformation. Examination of bone tissue and cartilage did not reveal any abnormal proliferation or pathological changes, affirming the benign nature of the tumor. Pathologists focused on evaluating the boundaries between these heterogeneous tissues, confirming clear demarcation without invasion into the surrounding normal tissues.

While this patient did not exhibit overt symptoms, typical or atypical symptoms of uterine teratoma may include but are not limited to abnormal uterine bleeding, abdominal or pelvic discomfort, dysmenorrhea, or palpable abdominal mass. Particularly in postmenopausal women, any new-onset uterine bleeding should raise high suspicion as it could signify underlying malignant pathology. However, symptoms are not always specific due to the rarity and varied presentations of uterine teratomas. Diagnostic criteria and processes for uterine teratoma typically commence with detailed medical history collection and physical examination, including gynecological examination. The initial diagnostic steps usually involve ultrasonography, a widely available non-invasive technique that provides preliminary information about the mass size, location, and structure. In ultrasound images, teratomas may manifest as complex masses containing solid and liquid components. Subsequent imaging evaluations, such as magnetic resonance imaging (MRI), are often required to obtain more detailed characteristics of the mass and its relationship with surrounding tissues. Hysteroscopy examination plays a crucial role in this diagnostic process, not only because it allows for visual assessment but also because directed biopsies can be performed. Biopsy and subsequent pathological analysis are the gold standard for confirming the diagnosis, as specific tissue types contained within the mass can only be determined through histological examination.

Furthermore, ruling out other potential etiologies is essential to the diagnostic process. For instance, differentiation between uterine teratoma and other types of uterine tumors, such as uterine fibroids, endometrial polyps, or possible malignant tumors, often relies on the radiographic features of the mass, pathological results and the patient’s clinical presentation. By integrating these diagnostic steps and methods, uterine teratoma can be effectively diagnosed, ensuring patients receive appropriate treatment and management.

## Discussion

Teratoma, one of the most common types of GCT, usually occurs in the gonads and is prevalent in infants and young children. Although ectopic teratomas are relatively rare, when they occur, they are often located in midline structures, accounting for about 1-2% of all teratomas. In particular, teratomas originating from the uterus have a very low incidence. These teratomas consist of tissues from all three germ layers and present with various symptoms; around 20% of cases may be asymptomatic (8). Furthermore, although the risk of malignant transformation is low, the potential for malignant transformation of mature cystic teratomas should not be ignored, especially in older women (9).In terms of diagnosis, ultrasound can typically provide an initial diagnosis of uterine teratomas, which can then be confirmed through postoperative histopathological examination. Imaging plays a crucial role in diagnosis, with typical ultrasound features such as a dermoid plug or Rokitansky nodule serving as strong indicators for teratoma diagnosis (8). The misdiagnosis risk of uterine teratomas mainly stems from their rarity and nonspecific clinical and imaging presentations; for example, in a case report of a 21-year-old Sukuma woman from northern Tanzania, the patient, who had been breastfeeding for eight months after a cesarean section, presented with symptoms of abdominal pain, bloating, fever, and abnormal vaginal discharge over the past three weeks. Initial pelvic ultrasound suggested a possible pelvic abscess, but subsequent exploratory laparotomy and histological analysis of the excised enlarged uterus ultimately confirmed the diagnosis of a mature uterine teratoma. This case underscores the importance of considering the possibility of uterine teratoma in patients with uterine masses in clinical practice, even without typical radiological findings (22). In postmenopausal women, any new uterine mass should raise concerns for clinicians, as it could be a sign of malignancy. However, due to the extreme rarity of uterine teratomas in postmenopausal women, clinicians may initially consider more common etiologies. We compiled previously reported cases of uterine teratomas to better understand the clinical management of uterine teratomas (**Table 1**). Moreover, when the mass shows features like fat-containing or complex solid-cystic structures, it may be misdiagnosed as other types of benign or malignant tumors (**Figure 5**). Regarding management strategies, the treatment approach is often determined based on the patient’s age, malignant risk, and fertility desires, with common treatment methods, including surgical excision, sometimes in combination with radiotherapy or chemotherapy if necessary (9).

**Table 1:** Uterine teratomas literature summary.

Journal	Authors	PMID	Year	Case Presentation	Diagnosis	Treatment	Outcome
Int J Mol Sci	Cong L, Wang S, Yeung SY, Lee JHS, Chung JPW, Chan DYL	37047114	2023	Ovarian dermoid cysts, most common in young women.	Diagnosed by ultrasound, confirmed by histopathology.	Depends on malignancy risk, patient age, and fertility requirements.	Outcomes vary with tumor type and treatment options.
J Obstet Gynaecol Res	Atwi D, Kamal M, Quinton M, Hassell LA	36053141	2022	Rare malignant transformation of ovarian cystic teratoma.	Diagnosed by tumor markers and imaging, with consideration of differential diagnoses.	Primary cytoreductive surgery, followed by chemotherapy and radiotherapy.	Outcome dependent on early detection and comprehensive treatment.
Eur J Orthop Surg Traumatol	Mavrogenis AF, Agrogiannis G, Toutouzas KG	35377080	2023	Primary immature teratoma of the thigh, an unusual location.	Classified as mature and immature based on the derivation from germ layers.	Not specified in the text.	Outcomes not specified.
Medicine (Baltimore)	Guo JX, Zhao JG, Bao YN	36596010	2022	Adult sacrococcygeal teratoma, very rare in adults.	CT and MRI for detection; diagnosis confirmed by surgical resection.	Surgical resection is the primary treatment.	Successful removal with surgery, watchful monitoring for malignancy.
J Obstet Gynaecol Res	Zhao Y, Xu T, Bu X, Yuan D, Wu Y, Qian H	33197962	2021	Immature teratoma in uterine corpus of an 11-year-old girl.	Initial diagnosis by ultrasonography, confirmed post second surgery.	Surgery followed by chemotherapy.	No recurrence during 2 years of follow-up.
Medicine (Baltimore)	Li M, Zheng W	38552061	2024	Rare case of mature solid teratoma in the uterine cervix.	Diagnosed by histological examination of the cervical mass.	Tumorectomy performed after discovering the mass.	No recurrence three months post-surgery.
Diagn Pathol	Ito K, Yano M, Ogasawara A, Miwa M, Kozawa E, Yasuda M	31684979	2019	Unique cervical 'teratocarcinosarcoma' resembling sinonasal TCS.	Diagnosed by biopsy showing carcinosarcomatous and teratomatous features.	Total hysterectomy with bilateral salpingo-oophorectomy followed by radiotherapy.	Disease-free for 13 months after treatment.
Int J Gynecol Pathol		27636886	2017	Simultaneous occurrence of immature and mature teratomas in the uterus and cervix.	Identified via histological examination showing immature and mature teratomas.	Initial surgery for endometrial and ovarian teratomas, followed by additional interventions.	Unique recurrence pattern suggesting a variant of growing teratoma syndrome.
Int J Gynecol Pathol	Wang WC, Lee MS, Ko JL, Lai YC	21979590	2011	Case of uterine mature cystic teratoma with unique origin.	Confirmed by CT and histological analysis, origin determined by DNA profiling.	Tumor resection followed by genetic analysis.	Determined the non-parthenogenetic origin of the uterine teratoma.
Int J Mol Sci	Cong L, Wang S, Yeung SY, Lee JHS, Chung JPW, Chan DYL	37047114	2023	Ovarian dermoid cysts, most common in young women.	Diagnosed by ultrasound, confirmed by histopathology.	Depends on malignancy risk, patient age, and fertility requirements.	Outcomes vary with tumor type and treatment options.
Pan Afr Med J	Bonahy AA, Sabbah H, Vadell AHM, Baba NEM	28819483	2017	Menopausal patient with malignant transformation within ovarian dermoid cyst	Malignant transformation within ovarian dermoid cyst	Surgery; in women of childbearing age and early disease stages, unilateral annexectomy without adjuvant therapy; in menopausal women, extensive surgery	Favorable
BMC Clin Pathol	Kamgobe E, Massinde A, Matovelo D, Ndaboine E, Rambau P, Chaula T	27011758	2016	21-year-old female from Tanzania with abdominal pain, distension, fever, and abnormal vaginal discharge for three weeks, lactating for 8 months post-cesarean, initially suspected pelvic abscess	Mature uterine teratoma confirmed after laparotomy and histological analysis	Initial pelvic ultrasound suggested pelvic abscess, followed by laparotomy and histological analysis of a bulky uterus	Favorable outcome

While researching uterine teratomas, we face a series of challenges. Uterine teratomas, although rare in clinical practice, demand high alertness and comprehensive judgment from doctors due to their complexity and diversity. Firstly, the diagnosis of uterine teratomas relies on a variety of medical imaging techniques. Uterine teratomas typically present as cervical lesions or polyps, which can lead to abnormal bleeding, uterine enlargement, and pain. However, in many cases, uterine teratomas may not exhibit specific symptoms, making preoperative diagnosis challenging. As stated in the literature (**Table 1**), ultrasound and imaging examinations play a crucial role in diagnosing mature cystic teratomas, being able to preliminarily identify the characteristics of teratomas in most cases (8).

Additionally, for suspected malignant transformations of mature teratomas, clinicians need to be highly vigilant, especially regarding potential changes in tumor marker levels and size (9). The manifestations of uterine teratomas vary among different age groups. In adult women, uterine teratomas may be discovered due to atypical symptoms such as vaginal bleeding or pelvic pain. These symptoms may be less obvious or misunderstood in children or adolescents, making the diagnosis more challenging. For instance, a study reported a case of primary immature uterine teratoma in an 11-year-old girl who had a history of abnormal vaginal discharge for six months. During an abdominal ultrasound, a solid mass was found in her uterus, leading to surgical treatment. A PET-CT scan performed 20 days postoperatively revealed a mass in her right ovary. Subsequently, the patient underwent a second surgery and received chemotherapy (18). This unique case of a young patient highlights the need for special attention to the potential rapid progression of the disease and long-term monitoring post-treatment. Another study reported the preoperative diagnostic experience of a 37-year-old woman with a mature uterine teratoma, emphasizing the importance of ultrasound and abdominal CT scans in the initial diagnosis (23). These imaging examinations help doctors accurately locate and assess the nature of the lesion before surgery.

In terms of treatment, in adult patients, depending on the size, type, and location of the tumor, treatment options may include conservative surgery (tumor resection only) or more aggressive methods (such as total hysterectomy). For younger patients, doctors typically opt for conservative treatment whenever possible to preserve fertility and minimize disruption to normal hormonal levels (24). In cases where further treatment is needed, adults may undergo chemotherapy or radiotherapy directly. However, in children and adolescents, these treatments may be approached more cautiously due to the potential impact on their growth and future fertility. Furthermore, in special types of uterine teratomas, such as uterine cervical “teratocarcinosarcoma,” their rarity and high invasiveness make them significant challenges in diagnosis. A case described in the literature indicates that this condition involves multiple tissue types and requires a comprehensive approach using surgery and radiotherapy for management (25). Although most uterine teratomas present as benign, their origins and developmental mechanisms are still not fully understood. Some studies suggest that uterine teratomas may differ in origin from ovarian teratomas, which is crucial for our understanding and treatment of these conditions (26). In this case, hysteroscopic resection was performed. Compared to traditional open surgery, hysteroscopic resection is a minimally invasive procedure that avoids large incisions, resulting in less postoperative pain and faster recovery. However, it may be less intuitive than open surgery for managing complex conditions (27, 28). In summary, hysteroscopic resection has become the preferred treatment for many gynecological diseases due to its minimally invasive nature, rapid recovery, and high diagnostic accuracy.

**Figure 6:**
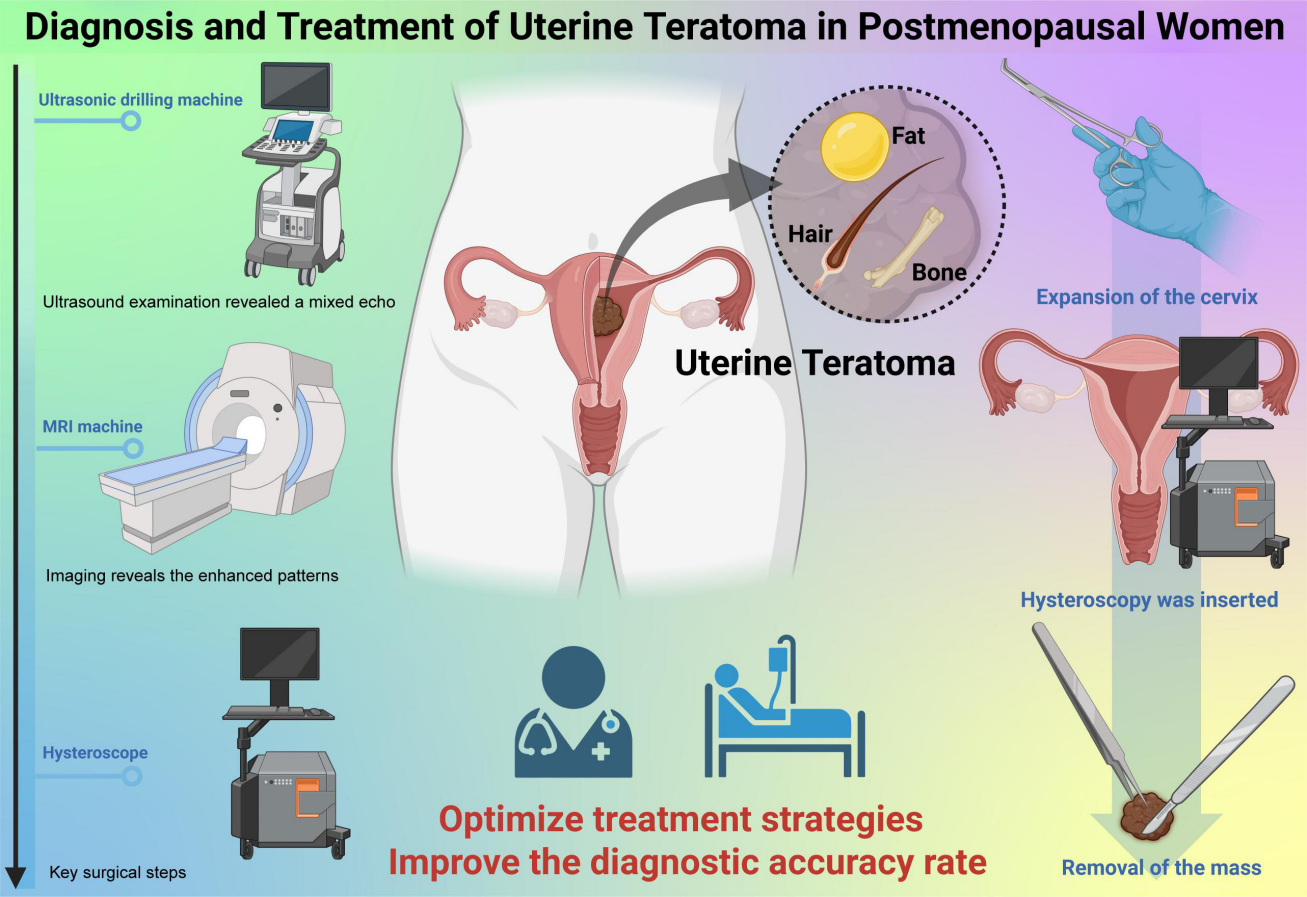
Diagnosis and Treatment of Uterine Teratoma in Postmenopausal Women.

In this patient’s case, no symptoms were exhibited throughout the treatment. The teratoma was only considered when the presence of fat and hair was discovered within the mass during hysteroscopy. Histologically, it was identified as a mature benign uterine teratoma. As the teratoma was located inside the uterine cavity and contained fat, it was initially mistaken for a submucosal fibroid with epithelial metaplasia based on vaginal ultrasound and tumor imaging. Despite its rarity, in women presenting with a uterine mass, a diagnosis of uterine teratoma should be considered, even when traditional ultrasound characteristics are absent. Due to its low incidence, no standardized management guidelines exist for mature uterine teratomas. Limited case reports suggest that total abdominal hysterectomy or complete tumor excision are the most effective treatment options (**Figure 6**).

## Data Availability

The original contributions presented in the study are included in the article/Supplementary Material. Further inquiries can be directed to the corresponding author.
